# The Maternal Effect Genes UTX and JMJD3 Play Contrasting Roles in *Mus musculus* Preimplantation Embryo Development

**DOI:** 10.1038/srep26711

**Published:** 2016-07-07

**Authors:** Lei Yang, Li-Shuang Song, Xue-Fei Liu, Qing Xia, Li-Ge Bai, Li Gao, Guang-Qi Gao, Yu Wang, Zhu-Ying Wei, Chun-Ling Bai, Guang-Peng Li

**Affiliations:** 1 The Key Laboratory of the National Education Ministry for Mammalian Reproductive Biology and Biotechnology, Inner Mongolia University, Hohhot, People's Republic of China; 2 State Key Laboratory of Natural and Biomimetic Drugs, Department of Chemical Biology, School of Pharmaceutical Sciences, Peking University, Beijing, People's Republic of China; 3 Department of Gynecology and Obstetrics, Inner Mongolia Medical University Affiliated Hospital, Hohhot, People's Republic of China

## Abstract

During the process of embryonic development in mammals, epigenetic modifications must be erased and reconstructed. In particular, the trimethylation of histone 3 lysine 27 (H3K27me3) is associated with gene-specific transcriptional repression and contributes to the maintenance of the pluripotent embryos. In this study, we determined that the global levels of the H3K27me3 marker were elevated in MII oocyte chromatin and decrease to minimal levels at the 8-cell and morula stages. When the blastocyst hatched, H3K27me3 was re-established in the inner cell mass. We also determined that H3K27me3-specific demethylases, UTX and JMJD3, were observed at high transcript and protein levels in mouse preimplantation embryos. In the activated oocytes, when the H3K27me3 disappeared at the 8-cell stage, the UTX (but not JMJD3) protein levels were undetectable. Using RNA interference, we suppressed UTX and JMJD3 gene expression in the embryos and determined that the functions of UTX and JMJD3 were complementary. When JMJD3 levels were decreased by RNA interference, the embryo development rate and quality were improved, but the knockdown of UTX produced the opposite results. Understanding the epigenetic mechanisms controlling preimplantation development is critical to comprehending the basis of embryonic development and to devise methods and approaches to treat infertility.

The maternal genome controls virtually all aspects of early animal development. Maternal mRNAs and proteins, which are loaded into the egg during oogenesis, implement basic biosynthetic processes in the early embryo, direct the first mitotic divisions, and specify initial cell fate and patterning^1^. As development proceeds, two processes are triggered that, together, form the maternal-to-zygotic transition (MZT) as follows: first, a subset of maternal mRNAs is eliminated, and second, the transcription of the zygotic genome begins^2^. Initially, maternally encoded products accomplish the destruction of maternal mRNAs. Terminally differentiated somatic cells can be reprogrammed to the totipotent state when transplanted into enucleated oocytes by means of somatic cell nuclear transfer (SCNT)^3^,^4^,^5^,^6^. When sperms or somatic cells enter the oocyte cytoplasm, they trigger epigenetic changes that eventually lead to the birth of viable animals. This action indicates the critical role played by the oocyte cytoplasm in embryonic development^7^,^8^. The mouse zygotic genome is activated at the 2-cell stage, which implies that embryonic development is transferred from the oocyte itself to the embryo^2^. Occasionally, mouse embryonic development is blocked at the preimplantation stage, although the mechanism for this inhibition is not clear^1^. Therefore, exploring the functions of some of the maternal factors in preimplantation embryos may help us to understand the potential reasons for early embryonic development failure.

Epigenetic mechanisms preside over our genetic information to enable development from the fertilized, totipotent zygote to the adult body. Many maternal proteins and mRNAs have been identified in mature murine oocytes and include variants of core histone proteins that associate with DNA to form the nucleosome^9^,^10^,^11^,^12^. Residue-specific methylation of histones is one of the most important epigenetic modifications and plays a crucial role in the transcriptional repression and activation of embryonic development – a process that involves both lineage specification and cellular differentiation. Many studies have demonstrated that epigenetic modifications reflect oocyte quality^13^. In mammals, two prominent histone modification markers are histone H3 Lysine 27 trimethylation (H3K27me3) and H3 Lysine 4 trimethylation (H3K4me3), which exhibit cell-dependent differential functions. Whereas H3K27me3 features silenced promoters, H3K4me3 is associated with active promoters. Interestingly, in cells of the inner cell mass (ICM), a large portion of genes modified by H3K27me3 are also marked by H3K4me3, and most of these so-called bivalent domain-containing genes encode transcription factors and signaling molecules of developmental importance^14^,^15^,^16^,^17^,^18^,^19^. The deposition and erasure of these histone marks are catalyzed by specific histone methyltransferases and demethylases. These observations have led to the suggestion that bivalent domains position genes in a poised state that allow for either timely activation or stable silencing in response to different developmental stages. H3K27me3 also regulates the expression of the three key pluripotency genes, Oct4, Nanog and Sox2, during the early differentiation of embryonic stem cells (ESCs). In addition, H3K27me3 is required for stem cell renewal and is considered to be important in maintaining pluripotency^20^.

The epigenetic regulation of gene expression is mediated primarily by trithorax group (trxG) proteins, which maintain the permissive chromatin state, and by polycomb group (PcG) proteins, which mediate a repressive chromatin configuration^21^. Two main PcG complexes, Polycomb Repressive Complex 1 (PRC1) and PRC2, exist in mammals^22^. The latter is primarily responsible for the generation of H3K27me3, causing gene repression, and it is primarily composed of the following three components: 1) the enhancer of zeste homolog 2 (EZH2)^23^,^24^; 2) the embryonic ectoderm development (EED) unit^25^; and 3) the suppressor of zeste 12 homolog (SUZ12)^26^. EZH2 is the functional methylase of H3K27me3, whereas EED and SUZ12 are cofactors required for EZH2 to perform this function^22^,^24^. Methyl residues are deposited at specific lysines by methylases and are removed by specific demethylases^27^. Hence, H3K27me3 can be demethylated, causing gene activation, by the JmjC domain-containing site-specific demethylases, UTX (ubiquitously transcribed TPR gene on the X chromosome, also known as KDM6A) and JMJD3 (Jumonji domain containing protein 3, or KDM6B)^28^,^29^,^30^,^31^.

UTX is a ubiquitously expressed protein that plays an important role in regulating the basal expression levels of H3K27me3 and the induced differentiation and development of the ectoderm and mesoderm^32^,^33^. Thus, UTX plays an essential role in somatic cell reprogramming^34^. In contrast, JMJD3 controls neuronal and epidermal differentiation and inhibits reprogramming^35^,^36^,^37^,^38^.

The homeotic (Hox) genes encode functionally critical transcription factors that modulate anterior-posterior pattern formation in the early embryo and assist in the regulation of embryonic segmentation^39^. During early embryonic development, UTX strictly regulates the temporal and spatial expression of Hox genes. A reduction in H3K27me3 levels is associated with the activation of many Hox genes^28^,^29^. It is unclear why demethylase proteins that antagonize polycomb-mediated repression, such as Jmjd3 and UTX, are expressed in terminally differentiated cells, where further changes in H3K27 methylation can be deleterious.

So far, the roles of UTX and JMJD3 in pre-implantation embryonic development are only understood in bovine species^40^. Moreover, in the classic mouse animal model, correlational studies have not been reported. We hypothesize that the global decrease in H3K27me3 expression observed after fertilization is due to the functional expression of JMJD3 and/or UTX, which both facilitates zygotic genome activation (ZGA) and enables embryonic development to proceed. To explore this hypothesis, we undertook parthenogenetic activation of mouse embryos in the present study. First, we determined the expression patterns of UTX and JMJD3 during the embryo preimplantation development. Second, we investigated the impact of knock-down of UTX and/or JMJD3 expression on H3K27me3 and the potential subsequent embryonic development.

## Results

### Dynamics of H3K27me3 during Preimplantation Development of Mouse Embryos

**Figure 1:**
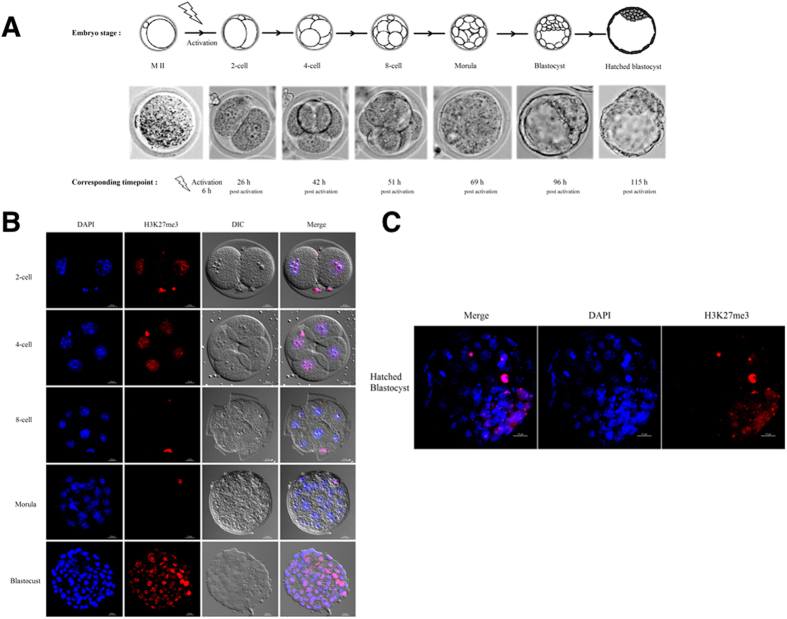
Experimental design and modification of H3K27me3 in preimplantation embryos. (**A**) Overview of parthenogenetically activated embryos and developmental stages in mice. (**B**) Parthenogenetic activation produced embryos at the 2-cell, 4-cell, 8-cell, morula, and blastocyst stages. Positive staining of H3K27me3 could be observed in the 2- and 4-cell stages. Global demethylation of H3K27 occurred at the 8-cell and morula stages. *Scale bar*, 20 μm. (**C**) A small proportion of cells was re-marked with H3K27me3 at the hatched blastocyst stage. *Scale bar*, 10 μm.

To detect H3K27me3 modification in the female nucleus of the mouse embryos, we employed an oocyte parthenogenetic activation method and collected the embryos at different time points (Fig. 1A). H3K27me3 modification was detected by immunofluorescence assay in GV, MII, 2-cell, 4-cell, 8-cell, morula, blastocyst and hatched blastocyst embryos (Fig. 1B). The results revealed that the modifications of H3K27me3 already exist in the MII oocytes. H3K27me3 in the 2-cell embryos is present at very low levels, but it can be detected. H3K27me3 cannot be detected at the 8-cell or morula stages. Subsequently, the H3K27me3 modification increased in whole blastocyst. When the blastocyst hatched, H3K27me3 was only detected in the ICM (Fig. 1C). It is worth noting that the polar body histone proteins always exhibited H3K27me3 modification, and the fluorescence signal was higher than in the embryo nucleus.

### H3K27me3 Demethylases *JMJD3* and *UTX* mRNA Abundance during Preimplantation Development

**Figure 2:**
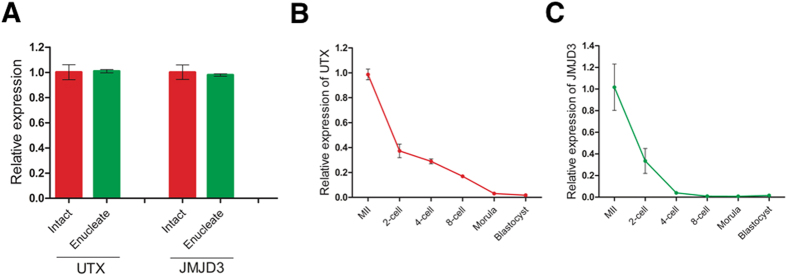
UTX and JMJD3 gene expression patterns in mouse oocytes and preimplantation embryos. (**A**) UTX and JMJD3 transcript levels in MII and enucleated oocytes. For all panels, the error bars indicate the means ± S.D. (n ≥ 3). UTX (**B**) and JMJD3 (**C**) transcript levels were determined by quantitative RT- PCR at different stages of preimplantation development. High levels of UTX and JMJD3 were observed in oocytes, and levels decreased in early embryos. Ct values of q-PCR analysis were normalized against GADPH. The data are represented as the means ± S.D. (n ≥ 3) from at least three independent experiments.

Because H3K27me3 exists as an “erase and rebuild” model in preimplantation embryos, this dynamic reconstruction process must be related to the catalytic enzyme. Real-time quantitative RT-PCR (RT-qPCR) demonstrated that the cytoplasm of mouse MII oocytes contained abundant levels of *UTX* and *JMJD3* transcripts (Fig. 2A). RT-qPCR identification of different stages of embryos showed that *JMJD3* and *UTX* mRNA transcript were abundance in the embryos, and both *UTX* and *JMJD3* transcripts began to degrade after activation and became almost undetectable in the blastocyst stage (Fig. 2B,C).

### Immunofluorescence and Immunoblot Analysis of UTX and JMJD3 Proteins in the Preimplantation Positioning Model

**Figure 3:**
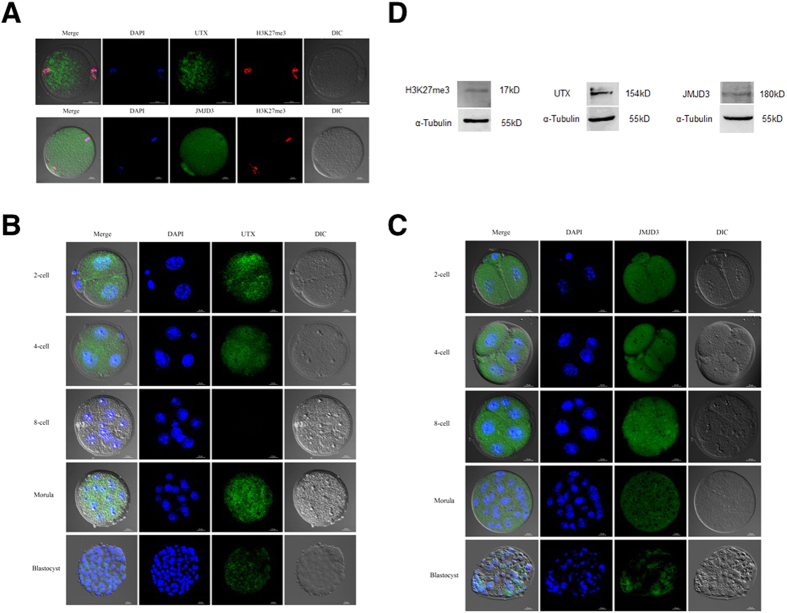
Subcellular distribution of UTX and JMJD3 in parthenogenetically activated preimplantation embryos. (**A**) Immunostaining of UTX and JMJD3 in MII oocytes. Upper panel, *scale bar*, 50 μm. Lower panel, *scale bar*, 20 μm. Immunofluorescence staining using anti-UTX (**B**) and anti-JMJD3 (**C**) antibodies at the 2-cell, 4-cell, 8-cell, morula, and blastocyst stages. *Scale bar*, 20 μm. (**D**) Immunoblot of MII oocytes with UTX, JMJD3 and H3K27me3 antibodies.

To determine the location of UTX and JMJD3 proteins in oocytes and parthenogenetic embryos, we performed immunofluorescence analysis using a commercial antibody raised against UTX and JMJD3 on oocytes and mouse embryonic fibroblasts (MEFs). UTX and JMJD3 proteins were present in abundance in the MII stage and distributed throughout the whole oocyte (Fig. 3A), and UTX was primarily distributed around the spindles (Fig. 3A). In the somatic cells, however, UTX was dominative concentrated in the nuclei of MEFs (Fig. S1). Importantly, the UTX protein was not detected in the 8-cell embryos, but the JMJD3 protein was present in this stage embryos (Fig. 3B,C). The disappearance of the UTX protein synchronized with the absence of H3K27 trimethylation, which indicated that UTX has other functions besides its classical demethylase function.

To further confirm the immunofluorescence staining results mentioned above, we collected more than 1,000 MII oocytes for SDS-PAGE and Western blot analysis (Fig. 3D). The results showed that bands detected at 154 and 180 kDa corresponded to UTX and JMJD3, respectively, which indicated that UTX and JMJD3 protein were present in the mouse MII oocytes.

### RNA Interference Technology Revealed that UTX and JMJD3 might Functionally Compensate for Each Other in the Mouse Embryos

**Figure 4:**
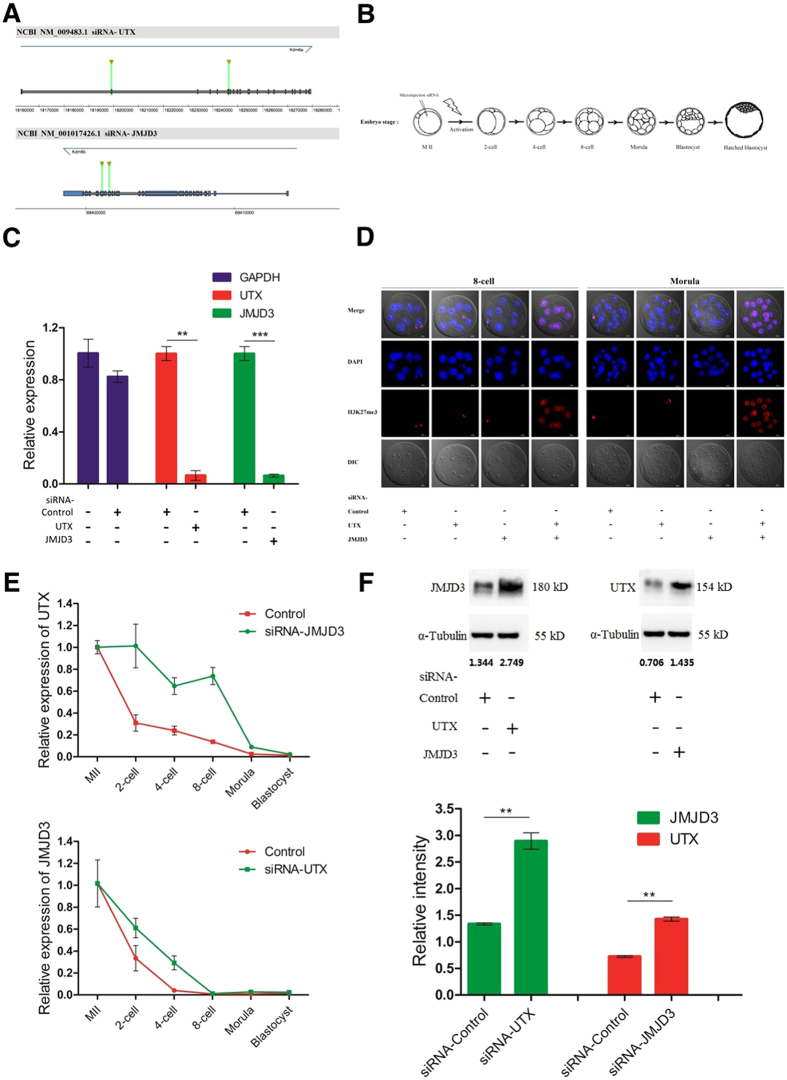
RNA interference technology reveals that UTX and JMJD3 can compensate for each other in the mouse preimplantation embryo. (**A**) Schematic representation of UTX and JMJD3 mRNA. Two siRNA species were designed to target different regions of UTX and JMJD3. (**B**) Schematic illustration of siRNA injection into oocytes and parthenogenetic activation. (**C**) Transcript analysis of UTX, JMJD3, and GAPDH levels after siRNA treatment and 96 h post-activation. siRNA-UTX and siRNA-JMJD3 oocytes were injected with siRNA as indicated. **P < 0.01, ***P < 0.001. (**D**) H3K27me3 immunostaining of 8-cell- and morula-stage embryos. Oocytes were injected with siRNA as indicated. DNA was stained with DAPI. The H3K27me3 levels between control embryos and embryos injected with either UTX or JMJD3 siRNAs cannot observe any difference, but a marked increase was observed when both siRNAs were injected, *scale bar*, 20 μm. (**E**) The dynamics of UTX and JMJD3 transcript levels in oocytes treated with siRNA 1 h before activation. Data are presented as the mean expression levels relative to GAPDH with siRNA-control injected oocytes normalized to 1. (**F**) Upper panel, we collected 2,000 oocytes in the 2-cell stage for immunoblotting analysis of JMJD3 and UTX proteins, respectively. Numbers below the western blots indicate band intensity (normalized to total α-Tubulin) measured by using ImageJ software. Lower panel, quantification of western blot results using scanning and ImageJ software. Results are expressed as integrated optical density. Each sample was normalized to α-Tubulin content, and the error bars indicate the means ± S.D. of triplicate values from a representative experiment, **P < 0.01.

UTX and JMJD3 are both histone demethylases with specific activities against H3K27me3. We speculated that UTX and JMJD3 functionally compensated in the oocytes. To verify this hypothesis, we designed and constructed siRNA specific target UTX and JMJD3 (Fig. 4A). To prevent the presence of UTX and JMJD3 alternatively spliced isoforms in oocytes, we designed two specific siRNA targeted at 585 and 1,518 bp downstream from the start codon, the two siRNA were then mixed to form siRNA-UTX. The similar protocol was used to construct two specific JMJD3 siRNA targeted at 4,536 and 4,749 bp downstream of the start codon, and mixed to create siRNA-JMJD3. A siRNA without any specificity against UTX and JMJD3 or any other gene in the genome was constructed as a control.

We microinjected UTX and JMJD3 siRNA into MII oocytes to validate their efficiency, specificity and induced parthenogenetic activation (Fig. 4B). RT-qPCR analysis of UTX or JMJD3 in the oocytes after siRNA injection demonstrated the knockdown efficiency of UTX and JMJD3. Both UTX and JMJD3 siRNAs reduced the UTX and JMJD3 mRNA levels over 90%, whereas the abundance of the controls did not altered (Fig. 4C). From the picture in Fig. 4D, we did not observe any difference in H3K27me3 levels between the control embryos and the embryos injected with either UTX or JMJD3 siRNA but a marked increase H3K27me3 levels were observed when both siRNA injected. The results indicate that the functions of UTX and JMJD3 are complementary.

Interestingly, when siRNA-UTX was injected, UTX mRNA expression decreased and JMJD3 mRNA expression increased. The same pattern was also observed in the JMJD3 interference group; the decrease in JMJD3 mRNA expression was accompanied by an increase in UTX mRNA expression (Fig. 4E). Immunoblotting also confirmed this phenomenon at the protein level as in Fig. 4F. These findings suggest that UTX and JMJD3 be functionally redundant and compensate for each other in early mouse embryonic development; upon interference of either UTX or JMJD3, the levels of the other transcript will increase.

### The Effect of UTX and JMJD3 on Parthenogenetic Embryo Development

**Figure 5:**
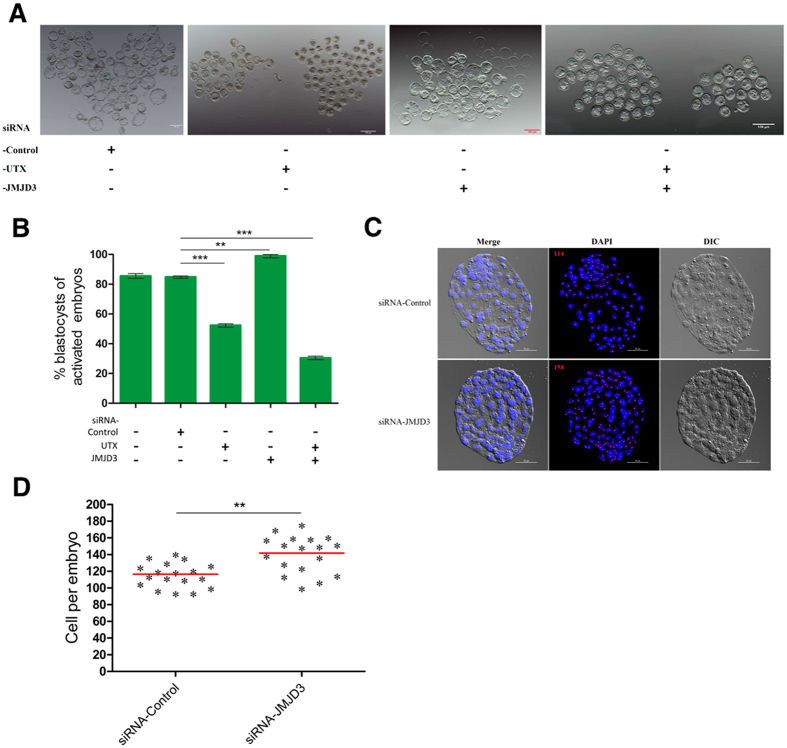
UTX or JMJD3 knockdown affects embryonic development of parthenogenetically activated embryos. (**A**) Representative images of siRNA injected embryos after activation 115 h of culture *in vitro*. *Scale bar*, 100 μm. (**B**) Blastocyst rate of siRNA-injected embryos after 115 h of culture *in vitro*. siRNA-Control, siRNA-UTX, and siRNA-JMJD3, oocytes were injected with siRNA as indicated. (**C**) Representative DAPI staining of blastocysts of siRNA-injected embryos after 115 hr of culture *in vitro*. *Scale bar*, 50 μm. (**D**) The blastocyst cell numbers were determined by counting the number of cells in DAPI-stained embryos. **P < 0.01, ***P < 0.001.

We used RNA interference technology to explore the role of UTX and JMJD3 in embryonic development, the results was shown in Table 1. Injection of siRNA-UTX in MII oocytes did not affect the cleavage of embryos. However, the proportion of embryos developed to the blastocyst stage was significantly lower in the siRNA-UTX-injected groups (52.7%) than in the control-siRNA-injected groups (84.3%) (Fig. 5A,B). Of particular interest, knockdown of JMJD3 significantly increased the blastocyst development rate (95.1%) (Fig. 5B). In consistent with our expectations, double knockdown of maternal UTX and JMJD3 in oocytes resulted in significantly compromised blastocyst development.

To further evaluate the quality of the disrupted blastocysts, we counted the number of cells in the blastocyst after single or double knockdown of UTX and JMJD3 (Fig. 5C). In these assays, JMJD3-siRNA-treated groups exhibited a higher total cell number than the blastocysts from the control-siRNA-injected groups (140.7 *vs.* 116.3 cells per embryo, respectively; *P* < 0.01; Fig. 5D).

### Knockdown of JMJD3 Promoted Blastocyst Expression of Oct4-GFP

**Figure 6:**
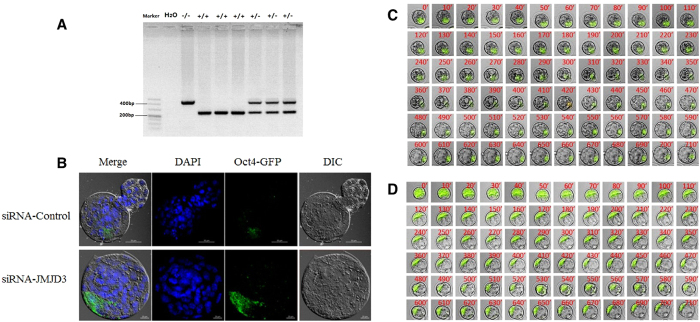
Knockdown of JMJD3 promoted blastocyst expression of Oct4-GFP. (**A**) PCR analysis of the Oct4-GFP allele. Genomic DNA was amplified with primers detecting the wild-type allele (upper panel) and primers spanning the Oct4-GFP region (lower panel). (**B**) Detection of Oct4-GFP expression in blastocysts, injected siRNA-JMJD3 or siRNA-control. Upper panel, *scale bar*, 50 μm. Lower panel, *scale bar*, 20 μm. Typical time-lapse images of Oct4 promoter-driven GFP dynamics during preimplantation development. Representative images of siRNA-control (**C**) and siRNA-JMJD3 (**D**) injected embryos. Selected images are shown at 10-min intervals from an acquired series. The time at which the observations were started was the morula stage. Original magnification, × 200.

Oct4 (Pou5f1), a key reprogramming factor and marker of pluripotency, is expressed in the ICM of the blastocyst stage, which is the gold standard for the identification of embryo quality. To prevent the loss of the Oct4-GFP gene during oogenesis, we used a nested PCR method to identify homozygous donor mice (Fig. 6A). To detect embryo quality by means of Oct4 promoter-driven GFP, we injected siRNA-JMJD3 or control-siRNA into OG2 parthenogenetic embryos and imaged the embryos via confocal laser-scanning microscopy (Fig. 6B) and live-cell imaging (Fig. 6C,D & Supplementary Movie). Compared with the control group, the GFP fluorescence signals significantly enhanced in the oocytes injected with JMJD3 siRNA.

## Discussion

The transition of a highly differentiated oocyte into totipotent blastomeres is a fundamental process during mammalian early embryo development^7^. Positive or negative regulation of particular factors must be involved in the transition. During the course of early embryogenesis, the temporal and spatial expression patterns and the corresponding functions of particular regulatory factors serve crucial roles in subsequent embryo development^2^. In vertebrates, the mature oocytes contain abundant proteins and nucleic acids, some of them persist up to the blastocyst stage and may involve the regulation of the initial events during early embryo development^2^. The discovery of histone methylation in 1960s, it was originally thought that histone protein methylation was irreversible^41^. In 2004, lysine-specific demethylase 1 (LSD1, also known as KDM1A) was identified as a demethylase capable of removing the histone lysine H3K4 mono-/di-methylation modification (H3K4me1/2)^42^,^43^. Soon later, the first JmjC domain-containing protein KDM2 which demethylates histone H3K36 was identified in 2005^44^. These studies indicate that histone protein methylation is reversible and the pattern of histone methylation is a dynamic process. Currently, the lysine demethylation enzymes such as LSD and JMJC domain families are known to have decisive effects on the early embryo development^43^.

Especially, the H3K27me3 is known to dominate repress genes’ expression, although certain genes are expressed irrespective of the occupancy of the H3K27me3 promoter^45^. This epigenetic modification plays important regulations in modulating both maintaining and differentiation of ESc^7^. The decrease in H3K27me3 levels in preimplantation embryos has been reported in different species, but the mechanism involved in it was unknown. In the present study, we immunofluorescent stained the nuclei of various stages of embryos and observed that the H3K27me3 were positive in the 2-cell and 4-cell stage embryos, and disappeared when the embryos reached the 8-cell and morula stages. These results indicated that the global demethylation of H3K27me3 occurred at the 8-cell and morula stages, which is inconsistent with a previous report^46^. These contrasting findings are probably due to the result of collecting the embryos at different time points in the studies. Subsequently, the whole embryo was marked with H3K27me3 at the blastocyst stage; however, asymmetric H3K27me3 was observed at the hatched blastocyst stage. The fact that the fluorescence signal of H3K27me3 was very weak in 2-cell stage embryos notably might be related to zygotic gene activation^7^. Previous studies have demonstrated that no corresponding enzyme activity of H3K27 trimethylation is observed in the polar bodies^47^, but we found the H3K27me3 persisted in the polar bodies from oocytes to blastocysts in this study. In the early blastocysts, we observed the H3K27me3 marker distributed both in the ICM and the trophoblast cells. The H3K27me3 modification was mainly concentrated in the ICM in the hatched blastocyst. These results are in consistent with the recent reports^14^,^18^,^20^. In those reports, a large set of developmental important genes in the ICM are H3K27me3 and H3K4me3 (*i.e.*, bivalent domains) enriched and these are repressive and activating histone modifications, respectively.

The JmjC domain-contained related proteins UTX and JMJD3 have proved to catalyze H3K27me3/2 demethylation^29^. The present study was the first systematic report in the localization patterns of both UTX and JMJD3 in mouse oocytes and preimplantation embryos. The immunofluorescent staining results demonstrated that the UTX was mainly concentrated in the nuclei in the MEFs. The JMJD3 distributed in both the cytoplasm and the nuclei in the MEFs, which is different from the results in 3T3 and HeLa cells^48^,^49^. This difference probably is due to as-yet unidentified functions of UTX and JMJD3 rather than the specific removal of methyl groups.

It is known that UTX and JMJD3 play complex roles in cancer pathogenesis, both of them are considered tumor suppressors. UTX regulates Rbl2 (retinoblastoma-like protein 2)-dependent cells fate control, and JMJD3 participates in oncogene-induced senescence by the activation of the *INK4b-ARF-INK4a* locus^49^,^50^. In the acute lymphoblastic leukemia, however, UTX and JMJD3 are reported the opposite functions in leukemogenesis^51^. The present data showed that the UTX protein appeared a "scattered point" distribution in the oocytes and preimplantation embryos, while the distribution of the JMJD3 protein was different from the UTX. In the 8-cell stage embryos, both of the H3K27me3 and UTX signals disappeared. The potential mechanism in the decrease of H3K27me3 during embryonic cleavage probably is the dilution of methylated marks with the continuation of cell divisions in which new histone incorporations occurred in the absence of PCR2 complex activity.

The present results indicated that although the preimplantation embryos had high UTX and JMJD3 protein expressions, the H3K27 remained in the trimethylated state. Therefore, we speculated that there might exist a protective mechanism for the modification of H3K27me3, which is similar to the DNA methylation to protect the H3K27me3 modifications in the early embryos^2^. The MZT is the first step for embryonic development. In the present study, the RT-qPCR and immunoblotting assay proved that significant levels of UTX and JMJD3 transcripts and proteins are stored in the cytoplasm of the MII oocytes. Upon the completion of activation, the UTX and JMJD3 mRNAs began to degrade and initiated ZGA. To further explore the functional expression of UTX and JMJD3 during early embryo development, we designed and constructed the UTX and JMJD3 specific siRNAs to target their transcripts. When the siRNA-UTX was injected alone, the endogenous oocyte JMJD3 transcription increased. Similarly, the only injection of siRNA-JMJD3 resulted in endogenous UTX increase. These results indicated that the knockdown of either UTX or JMJD3 increased the expression of JMJD3 or UTX, which suggested that there a feedback regulatory mechanism exist between the UTX and JMJD3. As the UTX and JMJD3 transcripts contain unique untranslated regions that possess novel regulatory elements, we try to figure out whether UTX or JMJD3 function equivalently during early embryonic development. The results showed that no matter the UTX or the JMJD3 was knocked down, the activated oocytes could develop to the blastocyst stage, which suggested that UTX and JMJD3 be functionally redundant and be capable of compensating for each other. However, when knockdown of both UTX and JMJD3, the embryos did not develop up to the blastocyst stage. In addition, the injection of siRNA-JMJD3 significantly improved not only the blastocyst development but also increased the blastocyst cell numbers. However, the injection of siRNA-UTX alone, the blastocyst development and the cell number significantly decreased.

In the somatic cells, the JMJD3-deficient MEFs produced significantly more induced pluripotent stem cells (IPSc) colonies than that in wild-type cells, whereas the ectopic expression of Jmjd3 markedly inhibited reprogramming^38^. These observations indicate that JMJD3 is a potent negative regulator in. IPSc and the inhibitory effects of JMJD3 are produced by both histone demethylase-dependent and histone demethylase-independent pathways. When both of the UTX and JMJD3 were knocked down, the embryo development was severely hampered, which also suggested that H3K27me3 demethylation be responsible for UTX and JMJD3 regulation. However, although the siRNA used in our study exhibits a highly efficient and specific interference effect, it cannot achieve complete knockdown of UTX and JMJD3. The UTY (ubiquitously transcribed TPR gene on the Y chromosome), another different member of the conserved JmjC subfamily with 84% homology to UTX probably contribute to this effect^52^,^53^. To further demonstrate the knockdown of JMJD3 improve the resulted blastocyst quality, we prepared an Oct4-GFP transgene (OG2-mice) to monitor Oct4 promoter activity via GFP expression^54^ following JMJD3 knockdown. Live-cell imaging vividly demonstrated that the Oct4-GFP signals in the siRNA-JMJD3 resulted blastocysts was significantly higher than that in the control group. The GFP expression was primarily concentrated in the ICM. The similar results were obtained by laser scanning confocal microscope (LSCM).

To our best of our knowledge, this is the first report to discuss the action of the UTX, JMJD3, and H3K27me3 during early embryonic development. The UTX or JMJD3 mRNA and their proteins are stored in oocytes as maternal sources and immediately degrade with the parthenogenetic activation of the oocytes. The knockdown of JMJD3 significantly improved the blastocyst quality, while the double depletion of UTX and JMJD3 resulted in reduced embryo development. In conclusion, the UTX and JMJD3 as maternal transcripts play critical roles during embryonic genome activation and blastocyst development. Precisely how UTX and JMJD3 improve blastocyst development and embryo quality is currently unknown and will need to be determined in the future studies.

## Methods

### Ethics Statement

All studies adhered to procedures consistent with the National Research Council Guide for the Care and Use of Laboratory Animals and were approved by the Institutional Animal Care and Use Committee at Inner Mongolia University.

### Chemicals

Chemicals were purchased from Sigma Chemical Co. (St. Louis, MO) unless otherwise indicated. Primers were synthesized by Takara Biotechnology Dalian Co. Ltd (Dalian, China). Antibodies were purchased from Merck Millipore Biotechnology Inc. (Merck Millipore, USA).

### Animals

C57BL/6N (B6), DBA/2 and (C57BL/6N × DBA/2) F1 (BDF1) strains of mice were purchased from Vital River Laboratories (Beijing, China). In order to detect reprogramming by means of Oct4 promoter driven GFP, BDF1 mice were replaced with OG2 mice that carry an Oct4-GFP transgene (JAX stock number 004654). All mice were reared in house under specific-pathogen-free conditions and were housed under controlled lighting conditions (light: 08:00~20:00). The animals had free access to food and water. The mice were randomly allocated to each experimental group.

### Collection of oocytes

Six to 8-week-old B6D2F1 female mice were used as metaphase II (M II) oocyte donors. In order to detect reprogramming by means of Oct4 promoter driven GFP, BDF1 mice were replaced with OG2 mice that carry an Oct4-GFP transgene (JAX stock number 004654). For *in vivo* MII stage oocyte-cumulus complex (OCC) collection, mice were superovulated by intraperitoneal injection of pregnant mare serum gonadotropin (PMSG; Sansheng, Ningbo, China, 10 IU) and human chorionic gonadotropin (hCG; Sansheng, Ningbo, China, 10 IU) 48 hours apart. Mice were sacrificed by cervical dislocation and MII oocytes were collected from oviducts 14 hours post hCG. In some experiments, cumulus cells were dispersed by 0.3 mg/ml hyaluronidase in HEPES-M2 medium.

### Oocyte parthenogenetic activation and *in vitro* development

The denuded oocytes were rinsed gently in Ca^2+^-free KSOM medium. Oocytes injected with siRNA and the negative control were activated with 10 mM SrCl_2_ and 5 μg/ml Cytochalasin B in Ca^2+^-free KSOM for 5 h at 37 °C in 5% CO_2_ in air. The activated oocytes were incubated in 20 μl drops of KSOM covered with mineral oil at 37 °C in 5% CO_2_ containing air. The embryos were checked at 26, 42, 51, 69, 96 and 115 hours post parthenogenetic activation to record the 2-cell, 4-cell, 8-cell, morula, blastocyst and hatched blastocyst stage, respectively.

### siRNA construction and microinjection in oocytes

Two different siRNA species targeting JMJD3 or UTX were designed and synthesized using the silencer siRNA construction kit (Ambion, USA) following the manufacturer’s instructions. The siRNAs were diluted to 20 μM in water and stored at −80 °C until use. A commercially available siRNA without any specificity to known genes was used as control. As previously described, with minor modifications, 8 pL of UTX and/or JMJD3 siRNA, control siRNA (20 μM) was microinjected into the cytoplasm of denuded MII oocytes. A 2 μL drop of siRNA was placed in the dish to fill the micropipette, and injections were performed using an inverted microscope (Ti-U, Nikon, Japan) equipped with micromanipulation equipment (Narishige, Japan) at room temperature. Oocytes were injected using a beveled Piezo-operated micro capillary needle micropipette (3~5 μm internal diameter), loaded with Fluorinert, using hydraulic microinjection equipment (Eppendorf, Germany). After injection, oocytes were kept at room temperature for 30 min and then moved into the incubator for at least another 30 min before parthenogenetic activation.

### RNA extraction and reverse transcription

As previously described, total RNA was extracted using the Pico-Pure RNA Isolation Kit (Arcturus, USA) according to the manufacturer’s instructions. Total RNA was extracted from each pool of oocytes/embryos (n = 3 pools of 30 oocytes or embryos per time point), and residual genomic DNA was removed by DNase I digestion, using an RNase-Free DNase Set (Qiagen, Germany). Reverse transcription was performed using SuperScript II (Invitrogen, USA) following the manufacturer’s instructions.

### RNAi analysis by real-time RT-PCR

Real time RT-PCR primers were designed and synthesized by Takara Biotechnology Dalian Co. Ltd. (Dalian, China). The *GAPDH* was used as an internal reference gene for normalization of *UTX* and *JMJD3* relative quantifications. The primers used are as follow:

*UTX*, forward-TATTGGCCCAGGTGACTGTGAA,reverse-CAGATCTCCAGGTCGCTGAATAAAC;*JMJD3*, forward-GCTGGAGTGCTTGTTCCATGAG,reverse-GAAAGCCAATCATCACCCTTGTC;*GAPDH*, forward-AAAATGGTGAAGGTCGGTGTG;reverse-AATGAAGGGGTCGTTGATGG

Real-time PCR was performed in an ABI prism 7500 Sequence Detection System. Each reaction mixture consisted of 2 μL of cDNA, 5 μmol each of forward and reverse primers, 7.5 μL of nuclease-free water, and 12.5 μL of SYBR Green PCR Master Mix (ABI, USA) in a total reaction volume of 25 μL. The steps were 95 °C for 10 min, 40 cycles of 95 °C for 15 s and 60 °C for 60 s. Dissociation curves were performed after each PCR run to ensure that a single PCR product had been amplified. Analysis of relative gene expression was measured using the 2 (-Delta Delta C (T)) method.

### Immunofluorescent staining

Oocytes were rinsed three times in phosphate-buffered saline (PBS) with 0.3% bovine serum albumin (BSA), fixed with 4% paraformaldehyde overnight at 4 °C, followed by permeabilization in PBS containing 0.3% vol./vol. Triton X-100 for 15 min followed by washing thoroughly in PBS containing 0.3% BSA. Fixed materials were blocked in 0.05% Twesen-20 in PBS containing 3% BSA overnight at 4 °C prior to the application of primary antibodies. After blocking and simultaneous incubating with rabbit polyclonal antibodies: anti-UTX (Millipore, USA) or anti-JMJD3 (Millipore, USA) for 1 h and goat polyclonal antibody anti-H3K27me3 (Abcam, USA) for 1 h at 37 °C. After washing with PBS containing 0.1% vol./vol. TritonX-100, the embryos were incubated with a secondary antibody conjugated with Alexa Fluor 594 and Alexa Fluor 488 (Invitrogen, USA) for 1 h at room temperature. For imaging the embryos were mounted in 5 μL anti-fade solution (ProLong Gold with DAPI, Invitrogen, USA) and compressed with a coverslip. After mounted on glass slides and examined with a Confocal Laser-Scanning Microscope (A1R, Nikon, Japan).

### Immunoblotting analysis

Immunoblotting was based on procedures previously reported. Briefly, oocytes were treated in sodium dodecyl sulfate (SDS) buffer and heated at 100 °C for 5 min, then cooled rapidly for 5 min. The proteins were separated by SDS polyacrylamide gel electrophoresis and electrically transferred to polyvinylidene fluoride membranes. Following transfer, the membranes were blocked in TBST (TBS containing 0.1% Tween 20) containing 5% non-fat milk at 4 °C overnight, followed by incubation at 4 °C for 12 h with either 1:2,000 anti-mouse-α-Tubulin antibody or 1:800 rabbit polyclonal anti-UTX or JMJD3 antibody. After washing 3 times in TBST, 10 min for each washing, the membranes were incubated for 2 h at 37 °C with 1:1,000 horseradish peroxidase-conjugated goat anti-mouse IgG or horseradish peroxidase-conjugated goat anti-rabbit IgG. The membranes were then processed using an enhanced chemiluminescence detection system (Thermo, USA).

### Live-cell Imaging of Oct4-GFP

Embryos produced by injected siRNA-JMJD3 or siRNA-control of OG2 oocyte were imaged on the stage of a Nikon Ti-E inverted microscope fitted with a Nikon A1R camera system. Laser scanning confocal microscope with 488 nm excitation for GFP.

### Statistical Analysis

Independent t-tests were performed using SPSS software version 22.0 (SPSS Inc., Chicago, IL, USA) to compare difference between two groups. The multiple comparison tests were analyzed by ANOVA using SPSS software followed by the Newman-Keuls test^55^. Differences at *P* < 0.05 were considered statistically significant.

## Additional Information

**How to cite this article**: Yang, L. *et al*. The Maternal Effect Genes UTX and JMJD3 Play Contrasting Roles in *Mus musculus* Preimplantation Embryo Development. *Sci. Rep.* **6**, 26711; doi: 10.1038/srep26711 (2016).

## Supplementary Material

Supplementary Movie S1

Supplementary Movie S2

Supplementary Information

## Figures and Tables

**Table 1:** Preimplantation development rates of embryos injected with siRNA, related to Figure 5B.

Injected siRNA-	No. of injected MII oocytes	No. of cleaved (2-cell) (% of injected oocytes)	No. of 4-cell embryos (% of injected oocytes)	No. of morulae (% of injected oocytes)	No. of blastocysts (% of injected oocytes)
—	200	200 (100.0)^a^	197 (98.5)^a^	186 (93.0)^b^	173 (86.5)^b^
Control	400	392 (98.0)^a^	389 (97.3)^a^	366 (91.5)^b^	337 (84.3)^b^
UTX	520	509 (97.9)^a^	365 (70.2)^b^	307 (59.0)^c^	274 (52.7)^c^
JMJD3	634	625 (98.6)^a^	624 (98.4)^a^	612 (96.5)^a^	603 (95.1)^a^
UTX + JMJD3	332	296 (89.2)^b^	226 (68.1)^b^	193 (58.1)^c^	103 (31.0)^d^

Data were analyzed using the Newman-Keuls test.

Values in the same column with no common superscript letters are significantly different (*P* < 0.05).
