# Supplementary material for: The Maternal Effect Genes UTX and JMJD3 Play Contrasting Roles in Mus musculus Preimplantation Embryo Development
Source: Sci Rep. 2016 Jul 7;6:26711. doi: 10.1038/srep26711 (PMC4935995; doi:10.1038/srep26711)
Supplement: Supplementary Information [file srep26711-s3.pdf]

# The Maternal Effect Genes UTX and JMJD3 Play Contrasting Roles in *Mus musculus* Preimplantation Embryo Development

Lei Yang[1], Li-Shuang Song[1], Xue-Fei Liu[1], Qing Xia[2], Li-Ge Bai[1], Li Gao[1], Guang-Qi Gao[1], Yu Wang[3], Zhu-Ying Wei[1], Chun-Ling Bai[1], and Guang-Peng Li[1,*]

[1]The Key Laboratory of the National Education Ministry for Mammalian Reproductive Biology and Biotechnology, Inner Mongolia University, Hohhot, People's Republic of China.

[2] State Key Laboratory of Natural and Biomimetic Drugs, Department of Chemical Biology, School of Pharmaceutical Sciences, Peking University, Beijing, People's Republic of China.

[3] Department of Gynecology and Obstetrics, Inner Mongolia Medical University Affiliated Hospital, Hohhot, People's Republic of China.

* Correspondence and requests for materials should be addressed to Guang-Peng Li (Email: gpengli@imu.edu.cn)

Supplement figure：

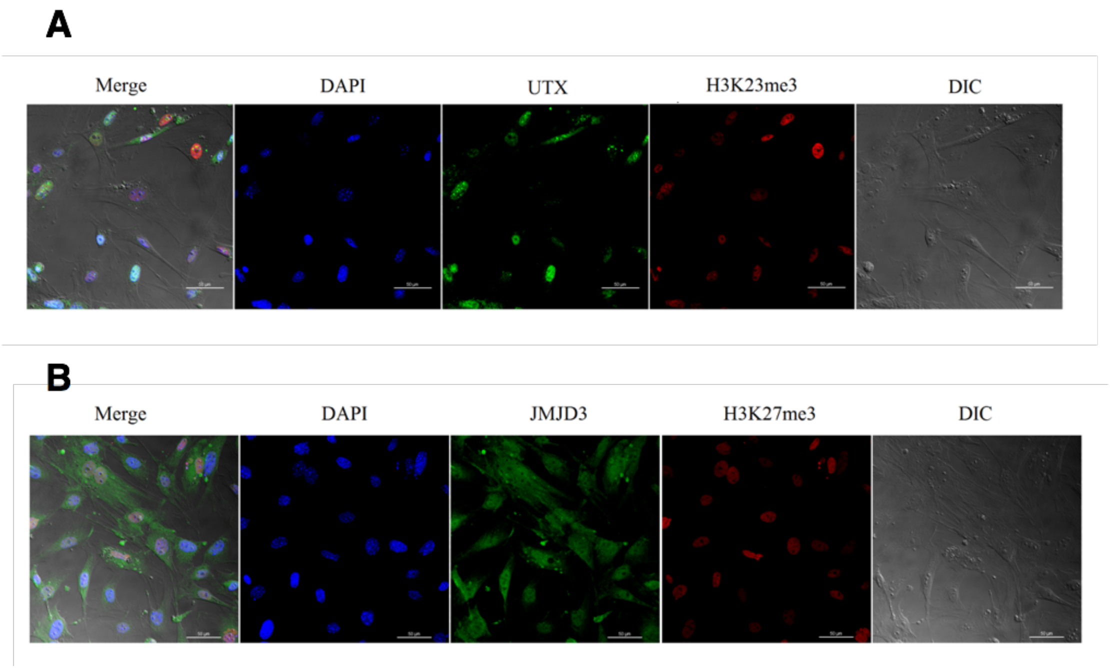


Figure s1. Immunostaining analysis for UTX (**A**) or JMJD3 (**B**) in mouse embryonic fibroblast cell. *Scale bar*, 50 μm.

Supplement movie：

To detect embryo quality by means of Oct4 promoter-driven GFP, we injected control-siRNA (Supplementary Movie1) or siRNA-JMJD3 (Supplementary Movie2) into OG2 (Oct4-GFP transgenic) parthenogenetic embryos and imaged via live-cell workstation. Compared with the control group, the GFP fluorescence signals significantly enhanced in the oocytes injected with JMJD3 siRNA.
